# The persistence of multiple strains of avian influenza in live bird markets

**DOI:** 10.1098/rspb.2017.0715

**Published:** 2017-12-06

**Authors:** Amy Pinsent, Kim M. Pepin, Huachen Zhu, Yi Guan, Michael T. White, Steven Riley

**Affiliations:** 1 MRC Centre for Outbreak Analysis and Modelling, Department of Infectious Disease Epidemiology, School of Public Health, Imperial College London, London, UK; 2 National Wildlife Research Center, United States Department of Agriculture, Animal and Plant Health Inspection Service, Wildlife Services, Fort Collins, CO, USA; 3 Joint Influenza Research Centre (SUMC/HKU), Shantou University Medical College, Shantou, People's Republic of China; 4 State Key Laboratory of Emerging Infectious Diseases/Centre of Influenza Research, School of Public Health, The University of Hong Kong, Hong Kong, SAR, People's Republic of China

**Keywords:** avian influenza, critical community size, *R*_0_, co-infection, stochastic model

## Abstract

Multiple subtypes of avian influenza (AI) and novel reassortants are frequently isolated from live bird markets (LBMs). However, our understanding of the drivers of persistence of multiple AI subtypes is limited. We propose a stochastic model of AI transmission within an LBM that incorporates market size, turnover rate and the balance of direct versus environmental transmissibility. We investigate the relationship between these factors and the critical community size (CCS) for the persistence of single and multiple AI strains within an LBM. We fit different models of seeding from farms to two-strain surveillance data collected from Shantou, China. For a single strain and plausible estimates for continuous turnover rates and transmissibility, the CCS was approximately 11 800 birds, only a 4.2% increase in this estimate was needed to ensure persistence of the co-infecting strains (two strains in a single host). Precise values of CCS estimates were sensitive to changes in market turnover rate and duration of the latent period. Assuming a gradual daily sell rate of birds the estimated CCS was higher than when an instantaneous selling rate was assumed. We were able to reproduce prevalence dynamics similar to observations from a single market in China with infection seeded every 5–15 days, and a maximum non-seeding duration of 80 days. Our findings suggest that persistence of co-infections is more likely to be owing to sequential infection of single strains rather than ongoing transmission of both strains concurrently. In any given system for a fixed set of ecological and epidemiological conditions, there is an LBM size below which the risk of sustained co-circulation is low and which may suggest a clear policy opportunity to reduce the frequency of influenza co-infection in poultry.

## 1. Introduction

The transmission of avian influenza (AI) within live bird markets (LBMs) in South East Asia continues to threaten human and animal health [1,2]. LBMs are a known source of AI in which high densities of poultry, unhygienic conditions, infected drinking water and dust particles all serve to amplify transmission [3–6]. The continuous introduction of naive birds into LBMs replacing those sold ensures that the pool of hosts susceptible to infection is continuously replenished, facilitating ongoing transmission of AI within LBMs. In addition, the movement of humans and poultry between farms and markets across different geographical areas further contributes to the spread of multiple AI strains [3,5].

The critical community size (CCS) for a pathogen is defined as the threshold population size required for the persistence of transmission, such that the probability of stochastic fade-out is very low [7], and can be derived analytically for certain diseases for a given value of *R*_0_. While the concept has been used for infectious diseases of humans for many years [8] and also within wildlife populations [9] it has not been applied to the persistence of AI in a multi-strain system. The persistence of AI within LBMs is of public health and evolutionary importance; if multiple different AI strains persist in LBMs, there is greater opportunity for co-infection, where a co-infection is the presence of at least two independent viruses within a single host. Frequent co-infections with genetically distinct viruses increase the opportunity for novel viruses to be generated through reassortment.

The importance of understanding the dynamics of AI and risk factors for transmission in LBMs first became apparent following the emergence of a new reassortant virus, highly pathogenic AI (HPAI) H5N1 in poultry in 1996 [1], and the subsequent detection of human cases in 1997 [10]. More recently, the emergence of novel reassortant viruses including: H10N8 [11], H5N8 [12], H7N9 [13], and the subsequent spillover into the human population, justify the ongoing concern LBMs present to humans and poultry.

Reassortant viral lineages continue to be identified in routine surveillance data collected from LBMs [14,15]. Because co-infection of a host with two different strains is a necessary prerequisite for reassortment to occur, the higher the number of co-infection events that occur, the greater the chance that transmissible and pathogenic novel reassortant progeny will be generated. Here, we use the word strain to describe a distinct lineage that may reassort with another lineage. Currently, we have a poor understanding of the frequency of co-infection in LBMs and the factors driving persistence [16]. A better understanding of factors that predict the persistence of multiple strains would provide a foundation for advancing our ability to predict the risk of reassortment. Worryingly, an increasing number of studies are showing that reassortment between different AI subtypes and human endemic subtypes can readily occur [17,18] and spillover of AI infections into the human population continue to be reported as a consequence of exposure to LBMs.

Mathematical models that seek to capture the dynamic nature and rapid turnover of LBMs remain rare, with detailed work on the mechanisms that generate the observed dynamics of infection restricted to single strains [3,16]. One such analysis by Pepin *et al.* [16] suggested that components of the poultry distribution system (farms and wholesale LBMs (wLBMs)) feeding into retail LBMs (rLBMs) were likely to be important points in the supply chain that were contributing to the seeding and on-going spread of AI within LBMs, but that rLBMs alone were unlikely to be the source of all infection [16]. Therefore, further investigation is required at points in the supply chain prior to birds entering LBMs.

If effective public health control measures are to be developed it is important to understand the ecological and epidemiological drivers of persistence such that we understand the conditions in which AI persistence is most likely to occur. Here we use a multi-strain stochastic susceptible, exposed, infected, recovered (SEIR) model to investigate the drivers of single and co-infecting strains, where co-infecting strains are two genetically distinct, independent viruses within a single host. Subsequent reassortment that may occur following co-infection is not considered. We assess how different parameters impact the persistence probability of a single AI strain, we determine the CCS for single and co-infecting strains, and the relationship between the CCS, market turnover rate and the *R*_0_ of co-circulating strains. We highlight high-risk features of LBM systems that may increase opportunities for AI persistence. We then use time series data collected in Shantou, China, in 2006 to explore the mechanisms that may generate the non-persistent multi-strain dynamics observed in surveillance data from a wLBM.

## 2. Material and methods

### (a) Dynamic co-infection model

We modelled the dynamics of infection within a single LBM in rural China, where birds were moved into the LBM on a daily basis from different sized farms and geographical locations, and then moved out of the market on a daily basis through selling to other smaller markets, farmers or directly to individuals for consumption. Interactions and importations between external farms and the market were modelled such that at one time point within a day, birds would be brought into the market and at the same time point approximately the same proportion were sold onwards (i.e. instantaneous turnover). As turnover rates within markets remain poorly quantified, we also examined the effects of different fixed turnover rates (above and below 50%) on our model outcomes. We assumed the market population size remained approximately constant, which is the prevailing opinion of fieldworkers as physical space is a scarce resource within LBMs and is therefore highly utilized [19,20]. However, although the market population may be approximately constant across days (except at major holidays), it is unlikely to be constant throughout the course of a day—i.e. most birds come in in the morning (highest population size) and gradually leave throughout the day (population size decreases gradually throughout the day). Thus, we also evaluated how fluctuations in population throughout the day impacted CCS estimates. Insights from the variable population sizes throughout the day could be extrapolated to interpret the effects of variable population sizes during holiday seasons.

We developed a within-market probabilistic multi-strain SEIR Markovian model that allowed for co-infection with two strains [21,22]. The stochastic infection dynamics were governed by transitions between states depicted in the electronic supplementary material, figure S1. Infection was either transmitted by direct within-flock transmission or environmental transmission through direct contact with infected drinking water, faeces or fomites [3,21,23]. Parameter values are provided in the electronic supplementary material, table S1.

Birds in the *S* compartment were susceptible to infection with all strains (A, B and AB, which denote strain A, B and the co-infection with independent strains A and B (AB), respectively). Exposed (*E*) birds were infected but not infectious to other birds, and susceptible to infection with a second strain. *I* birds were infectious to other birds, and temporarily immune to infection with a second strain [24,25] as a consequence of the innate immune response to infection [26]. In terms of adaptive immunity, *R* birds were fully recovered, and immune to re-infection with the same strain (i.e. full homologous immunity) but were susceptible to infection with a second strain with the same probability as fully susceptible hosts (*S*) (no cross-immunity). States in which birds were *S*, *E*, *I* or *R* to both strains were denoted as subscripted state symbols. We do not explicitly model particular AI strains, but generally model possible multi-strain dynamics. Additional model description is provided in the electronic supplementary information. Co-infection here represents simultaneous infection with strain A and B, thus our notation for co-infection reflects the host's state. Supporting quantitative evidence for the relative infectivity of two strains in co-infected birds was limited, therefore, we assumed co-infected birds always transmitted strains A and B concurrently (i.e. equal fitness for both strains) and that this led to direct co-infection in a susceptible bird. As a minimum criteria for co-infection, each individual strain must be capable of establishing sustained transmission for a period of time in a rapid-turnover market. Therefore, we define *R*^total^_0_ for a single strain as the sum of *R*^wf^_0_ and *R*^env^_0_.

The contribution from the environment *R*^env^_0_ was2.1
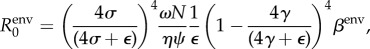
where *σ* is the duration of the latent period, *ε* is the rate at which birds are sold, *γ* is the duration of the infectious period, *ω* is the rate at which infectious birds shed virions, *N* is the number of birds in the market, *η* is the rate of virion decay in the environment, *ψ* is a restricted half-saturation constant for environmental transmission, and *β*^env^ is the transmission rate from the environment to birds.

The within flock *R*^wf^_0_ was calculated as:2.2
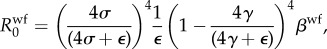
where *β*^wf^ is the transmission rate between birds.

Therefore, *R*^total^_0_ = *R*^wf^_0_ + *R*^env^_0_.

We note that an analytical derivation of an invasion criteria for the co-infecting strains in the system is not feasible here [21].

### (b) Assessing variation in the probability of persistence for a single strain

We assessed how variation in *β*^wf^, *β*^env^, *ω* and *η* (electronic supplementary material, table S1) impacted the probability of persistence for a single strain. We used Latin hypercube sampling of the four parameters (1000 samples) and calculated the partial rank correlation coefficient using ‘epiR’ [27] to determine if any parameters had substantially more impact on the probability of persistence.

### (c) Definition of a critical community size for the persistence of co-infecting strains

We define the CCS as the population size of the market required to ensure the persistence (at least one infected bird in the market) of a single strain 1 year after infection is seeded with a probability of at least 0.99 [7,8,28]. The dynamics of infection and circulating strains can vary markedly between different years, therefore we considered persistence only within a single year. The definition for the persistence of co-infecting strains is more complicated. Even if direct transmission of the co-infecting strain fades out, if both individual strains are still circulating, direct transmission of the co-infecting strains can re-emerge. Thus, we calculate the CCS for the persistence of the co-infecting strains by considering the frequency with which a single introduction of both strains separately results in an epidemic of the co-infecting strains which persists for at least 1 year, ensuring the number of birds infected with strain A and B (individually), or strain AB is greater than 1 at the end of the year. The CCS was estimated through simulation.

As variation in the population size directly impacted *R*_0_, we explored how the CCS varied when other components of *R*_0_ were altered. For the single-strain CCS, we increased the within-flock and environmental transmissibility, infectious period, viral decay and shedding rates to 25% and 75% above the baseline values. For the co-infecting strains CCS we decreased all aforementioned parameters by 25% for the co-infecting strains and then increased all aforementioned parameters for one of the founding strains by 75%. Sensitivity of CCS estimates for the co-infecting strains to changes in the duration of the latent period and market turnover rates were also assessed.

### (d) Surveillance data

Samples from named wLBMs in Shantou, China were taken between 2005 and 2006 and were collected reliably with a frequency of two to four weeks. Embryonated chicken eggs were used to isolate virus. Subtypes H1–H13 were tested for with monospecific antisera in haemagglutination inhibition (HI) tests. Further detail on the methods of data collection can be found in Pepin *et al.* [5].

**Figure 1.**
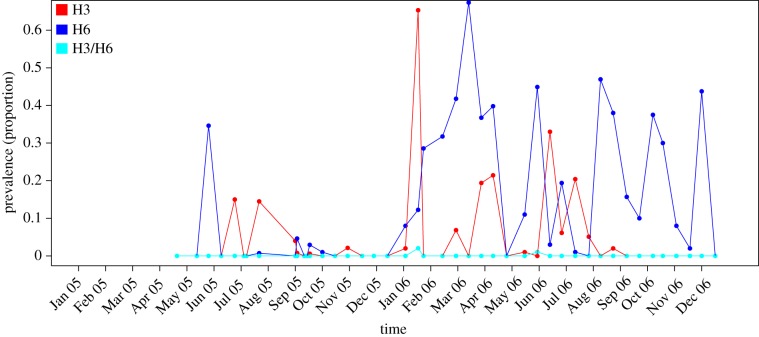
The prevalence of H3, H6 and H3/H6 co-infection in ducks in the surveillance data between 2005 and 2006. The red line shows the prevalence of H3, the blue shows the prevalence of H6 and the cyan line shows the prevalence of H3/H6 co-infecteds. Overall, the prevalence of both strains was much higher in 2006 compared with 2005, although despite this the overall prevalence of co-infection with both strains was low.

We focused on samples isolated from ducks from one wholesale market, as these were the hosts from which isolation of co-infected samples was highest [5], we looked at low pathogenic H3 and H6 infections (two strains from different subtypes), no data on neuraminidase type was available. Detail on the demography and ecology of the market was not collected, but in Shantou it is known that wholesale market birds come from both small backyard flock farms, as well as medium-sized poultry holdings where birds are kept indoors and isolated from other species [5]. Data from 2006 reported a higher prevalence of co-infection and larger single strain epidemic peaks when compared with 2005, therefore, we used data from 2006 (figure 1).

### (e) Seeding infection to generate the two-strain dynamics: comparison to surveillance data

We specified a random process for the introduction of each strain, where frequency, duration and prevalence of infection imported were drawn from random uniform waiting time distributions. Birds (5000) were brought into the market on a daily basis. Birds were imported on seeding days in the *I* state infected with strain A or B, all remaining birds were imported in the *S* state. An illustration of the seeding process is presented in the electronic supplementary material, figure S2. Maximum and minimum ranges of infection prevalence, and duration of seeding and non-seeding days were informed by the data. Each of the 10 models corresponding to different seeding regiments (electronic supplementary material, table S2) were run for 365 days, for 10 000 independent stochastic realizations. Co-infection data were not used to match the seeding scenarios owing to the very low reported prevalence.

Four summary statistics were calculated for each model realization and compared to those calculated from the data [29]. These were: (i) correlation between the two circulating strains; (ii) the number of epidemic peaks for each strain; (iii) the periodicity of the observed epidemics (by Fourier transform of the time series); and (iv) the mean prevalence of each circulating strain. Nonparametric approximations were used to locally estimate the probability density using the 10 000 realizations of each model for each summary statistic [29]. The model with the largest intersection of realizations across all four summary statistics that fell within ±50% of the data value was selected as the best performing model. All calculations and simulations were performed with R v. 3.2.1 [30].

## 3. Results

### (a) Persistence and critical community size for a single strain

**Figure 2.**
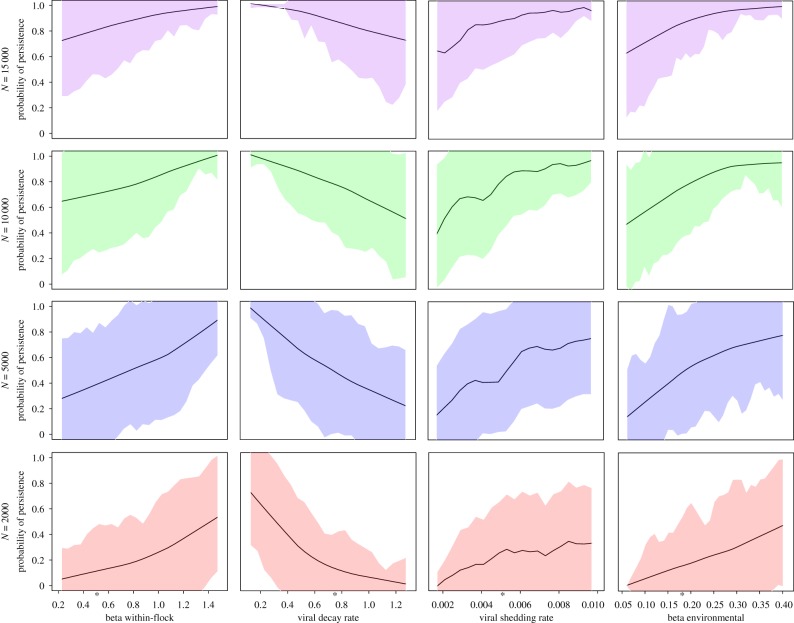
Probability of infection persistence for four key model parameters: *β*^wf^, viral decay rate (*η*), viral shedding rate (*ω*) and *β*^env^ across a range of values identified in the literature, considering a range of average population sizes from 2000 to 15 000 birds. We calculated the standard deviation of the mean for the probability of persistence for each range of parameter values provided, we used ± standard deviation of the mean to calculate the uncertainty about the mean for all parameters, indicated by the polygon. Baseline parameter values are indicated with an asterisk.

We explored the impact of four key model parameters on the probability of AI persistence (figure 2). Increases in the values of *β*^wf^, *β*^env^ and viral shedding rate (*ω*) increased the probability of persistence for a single strain, while increases in the rate of viral decay (*η*) decreased the probability of persistence. All parameter values are presented in the electronic supplementary material, table S1. Variation in the probability of persistence across the range of values evaluated reduced as the average daily population size of the market (*N*) increased. When *β*^wf^ was 0.2 and *N* = 2000 birds the probability of persistence was 0.05; when *N* = 15 000 the probability of persistence was 0.7. Thus, the probability of persistence increased by 1300% with a 650% increase in *N*. Equally, if *β*^env^ was 0.25 and *N* = 5000, the probability of persistence was approximately 0.60, but approximately 0.88 when *N* = 10 000 (a 46% increase in the probability of persistence with a 100% increase in *N*). Across all parameter ranges, the largest proportional increase in the probability of persistence was seen when *N* increased from 5000 to 10 000 birds (electronic supplementary material, figure S4).

At smaller population sizes, all parameters had a substantial impact on an invading strain's probability to persist (figure 2), generally, a steeper gradient and magnitude of change across the range of parameter values in smaller market sizes. When *N* was 5000 and *η* was 0.1, the probability of persistence was approximately 0.98, when *η* was increased to 1.2, the probability of persistence decreased to approximately 0.25 (74%). However, when *N* was 15 000 and *η* was 0.1 and the probability of persistence was 1. When the *η* was 1.2 the probability of persistence was 0.7 (a 30% decrease) (figure 2). Differences in the probability of persistence across the parameters were least substantial when looking at *η*, suggesting assumed values of viral decay rate may be a less important driver of persistence relative to the other parameters examined. By contrast, for any given *N*, the greatest variation in the probability of persistence was seen for *ω* (figure 2; electronic supplementary material, figure S4).

**Figure 3.**
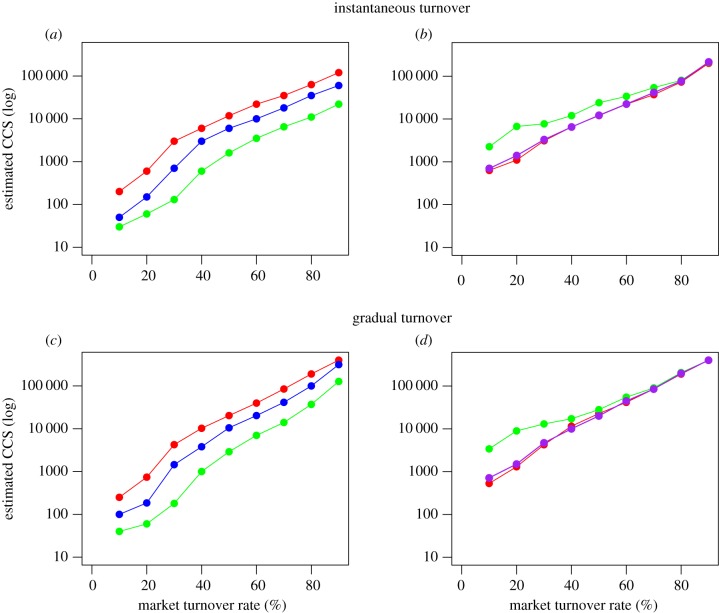
The CCS estimated through simulation analysis for 1000 realizations, for a range of market turnover rates. (*a*) Estimated CCS for a single strain across a range of instantaneous market turnover rates for the baseline parameters (red), when transmissibility of the strain was increased by 25% (blue), and by 75% (green). (*b*) Estimated CCS for the co-infecting strains across a range of instantaneous market turnover rates for the baseline parameters (red), when transmissibility of a single founding strain was increased by 75% (green), when the transmissibility of the co-infecting strain was reduced by 25% (purple). (*c*) Estimated CCS for a single strain across a range of gradual market turnover rates for the baseline parameters (red), when transmissibility of the strain was increased by 25% (blue), and by 75% (green). (*d*) Estimated CCS for the co-infecting strains across a range of gradual market turnover rates for the baseline parameters (red), when transmissibility of a single founding strain was increased by 75% (green), when the transmissibility of the co-infecting strains was reduced by 25% (purple).

Market turnover rate and hence average daily market population size played a key role in determining the probability of persistence. For our baseline parameters (electronic supplementary material, table S1) and an instantaneous market turnover rate of 50%, a sigmoidal relationship between the probability of persistence and CCS was observed (electronic supplementary material, figure S5*a*). Following increases in the population size above 11 800, no further marked increases in the probability of persistence were seen, suggesting the CCS for a single strain in this scenario was approximately 11 800 birds (figure 3*a*), with a corresponding *R*_0_ of ∼1.01 (electronic supplementary material, table S3). Assuming the baseline parameter values but where 50% of birds were sold gradually on a daily basis the estimated CCS was approximately 19 800 (figure 3*c*; electronic supplementary material, figure S5*c*), a 68% increase in the CCS compared with instantaneous turnover. As the market turnover rate increased, and hence the average duration of stay for a bird within the market decreased, the estimated CCS also increased (figure 3*a*). For our baseline parameters and an instantaneous market turnover rate of 10% (electronic supplementary material, table S1), the CCS was 200 birds (*R*_0_ ∼ 1.67), when 10% of the market population was sold gradually the CCS was approximately 250, a 25% increase in the estimated CCS. If an instantaneous market turnover rate of 90% was assumed the estimated CCS was approximately 120 000 birds, but if 90% were sold gradually the estimated CCS was approximately 400 000 birds (figure 3*b*), a 233% increase in the estimated CCS.

As the transmissibility, viral decay and shedding rates, and duration of infection for a single strain increased by 75% from the baseline values (electronic supplementary material, table S1), the CCS at any given rate of market turnover was reduced (figure 3*a*,*c*). However, if transmissibility of the strain was increased by 75%, the estimated CCS was approximately 3500 birds (*R*_0_ ∼ 1.32, electronic supplementary material, table S3) (figure 3*a*). As with the baseline parameters, estimates of the CCS with gradual turnover were consistently higher than with instantaneous turnover, however, the proportional differences in the CCS between turnover frequencies were consistent when parameter values were increased by 75% (figure 3).

### (b) Critical community size for the persistence of co-infecting strains

Assuming the baseline parameters (electronic supplementary material, table S1) and an instantaneous market turnover rate of 50%, the estimated CCS was approximately 12 200 (figure 3*c*; electronic supplementary material, figure S5*b*). As the average daily market population size decreased the probability of persistence of the co-infecting strains decreased. If 50% of birds were sold gradually throughout the day the estimated CCS was approximately 22 000 (figure 3*d*; electronic supplementary material, figure S5*d*), an 80% increase in the CCS.

With a 25% decrease in the baseline parameters of the co-infecting strains, the estimated CCS was similar to the baseline estimates assuming instantaneous turnover (figure 3*c*). When the instantaneous turnover rate was 60% or higher, the CCS for all three levels of transmissibility was similar, and tended to be approximately 500 birds greater than for a single strain (figure 3*c*,*d*). Generally, across all market turnover rates only a small increase in the CCS for the co-infecting strains was seen relative to the single strain whether turnover was instantaneous or gradual (figure 3*c*,*d*). This suggests that direct transmission of co-infecting strains is less important for the persistence of co-infection, and that the main factor facilitating the persistence of the co-infecting strains is the persistence of both individual strains, where sequential infection with both strains leads to a co-infection.

As the duration of the latent period increased for a fixed market turnover rate, the CCS of the co-infecting strains increased (electronic supplementary material, figure S6). Assuming a latent period of 6 days and a market turnover of 10%, the estimated CCS was approximately 1250 birds (electronic supplementary material, figure S6). However, if market turnover was increased to greater than 40%, the estimated CCS was greater than 100 000 birds. If the latent period was 3 days or less and market turnover was less than 75%, all CCS estimates were less than 100 000 birds (electronic supplementary material, figure S6).

As the transmissibility, decay and shedding rates and the duration of infection were increased by 75% for both founding strains, the average population size required to ensure persistence decreased (electronic supplementary material, figure S7). Considering a market turnover rate of 10%, the probability of persistence for 500 or more birds was 1, but if the baseline parameters were reduced by 25%, approximately 5000 birds were needed to ensure a persistence probability of greater than 0.99. If market turnover was 40% and baseline epidemiological parameters were increased by 75%, an average daily population of approximately 1000 birds ensured persistence. However, if market turnover was 70%, greater than 10 000 birds were needed to ensure a persistence probability of 1 (electronic supplementary material, figure S7).

### (c) Comparison with data

No evidence of persistent transmission of either of the two AI strains was observed in the surveillance data (figure 1), suggesting the population size of the market that generated the data was less than the CCS for a single strain identified in §3a. Therefore, an average population of 10 000 birds was assumed to evaluate 10 different seeding models outlined in the electronic supplementary material, table S2.

We initially found that autochthonous transmission was not sufficient to generate the observed infection incidence, and could not ensure that the regular outbreaks observed in the data were generated in the simulation (electronic supplementary material, figure S8).

Model 2 seeding a prevalence of 0–50% every 5–30 days, with a maximum non-seeding duration of 80 days was most consistent with the data. We judged consistency by the number of model realizations that were able to simultaneously match all the summary statistics (table 1). Models 1 and 2 scored the same for overall matching (6 out of 1000). We chose model 2 because model 1 had a very low number of matches to the number of strain 1 peaks. We present a random subset of four realizations obtained from model 2 (electronic supplementary material, figure S9). Models 1 and 5 were the next best performing. Both models drew prevalence values between 0 and 70% every 1–30 days, with a maximum non-seeding duration of 80 and 100 days, for models 1 and 5, respectively. Therefore, introduction of a relatively high prevalence of infection and a number of consecutive infection seeding days (greater than 5) are likely to be important features of the system.

**Table 1.** The model values are the density of observations that fell within the 95% confidence intervals of the data for each of the 10 models evaluated, for each summary statistic. (Higher values (up to 1) indicate that the model performed well for that summary statistic. Values provided in the data row represent the values for each summary statistic calculated from the data.)

model	correlation^a^	mean prevalence − strain 1^b^	mean prevalence − strain 2^c^	Fourier^d^	*N* peaks − strain 1^e^	*N* peaks − strain 2^f^	total matches^g^
data	−0.223	0.067	0.235	2.4	13	4	
model 1	0.52	0.33	0.95	0.40	0.08	0.37	0.006
model 2	0.41	0.11	0.66	0.15	0.71	0.24	0.006
model 3	0.54	0.41	0.62	0.15	0.25	0.20	0
model 4	0.001	0	0.99	0.88	0.20	0	0
model 5	0.18	0.15	0.49	0.18	0.31	0.05	0.005
model 6	0.003	0.40	0.001	0.01	0	0.24	0
model 7	0.03	0.14	0	0.02	0	0.10	0
model 8	0.02	0.13	0	0.01	0	0.11	0
model 9	0.003	0	0.99	0.47	0.72	0	0
model 10	0.07	0	0.99	0.83	0.64	0	0

^a^The correlation between the two strains circulating for the data and for all models the density of observations that fell within the 95% confidence intervals (CIs) of the data.

^b^The mean prevalence of the first strain and the density of stochastic observations that fell within the 95% CIs of the data.

^c^The mean prevalence of the second strain and the density of stochastic observations that fell within the 95% CIs of the data.

^d^The Fourier transform of the time series and the density of stochastic observations that fell within the 95% CIs of the data.

^e^The number of epidemic peaks of the first strain in the data and the density of stochastic observations that fell within the 95% CIs of the data.

^f^The number of epidemic peaks of the second strain in the data and the density of stochastic observations that fell within the 95% CIs of the data.

^g^Proportion of realizations where all statistics match together in that realization.

Models for which the range of seeding days was a maximum of 12 days (models 6–10), were unable to capture the calculated correlation between the two circulating strains. Models 9 and 10 had the shortest range of seeding and non-seeding days, where infection was seeded between every 1–12 days and not seeded every 2–40 days. This suggests frequent seeding and non-seeding intervals at high levels of prevalence may be able to capture the high prevalence of infection in the data (table 1), but not the dynamic interplay between two circulating strains. For models 6–10 the inability to capture some summary statistics resulted in none of these models being able to satisfy all criteria across the four statistics simultaneously (table 1). If the stringency of the criteria were reduced to assess the intersection of realizations between two or three summary statistics, very limited overlap between statistics for the same model were seen (electronic supplementary material, table S6).

## 4. Discussion

We have used a stochastic transmission model to investigate which mechanistic properties and parameter sets could capture prevalence data of two co-circulating strains seen in surveillance data collected from a wLBM in Shantou, China. Our findings highlight that re-introduction of infection into wLBMs is required to generate the infection dynamics seen in surveillance data. The need to continuously re-seed infection suggests that more needs to be done at the farming and production level to ensure that birds come to the market infection free.

Viral shedding rate and *β*^env^ were the most significant determinants of viral persistence, highlighting that further research should be conducted to more accurately quantify these parameters. The higher the market turnover rate the higher the average population size required for transmission to become self-sustaining, implying that both the size of the market and its expected turnover rate need to be considered when assessing the opportunities for AI persistence. This finding supports previous calls to reduce the time birds spend in the market [16,20]. Studies have shown that higher volumes of minor poultry sales resulted in a significant increase in virus isolation rates [20], and that live poultry market density was the most important predictor of H7N9 infection risk within poultry markets [31]. These findings in combination with the work presented in this article emphasize the importance of also considering market population size to limit the opportunity for AI persistence.

Depending on the geographical location of the market the estimates of the CSS identified across a range of parameter combinations may not be considered particularly large [32]. With increasing market turnover rate, the estimated CCS increased. Yet as the transmissibility of circulating strains increased, the CCS for a single strain reduced substantially. Our results show that persistence of co-infection under typical market conditions requires continued seeding of both strains, and that continual introduction of both strains separately (as opposed to introduction as co-infections) can lead to long-term persistence of co-infection. This observation may mean that the co-circulation and persistence of multiple strains increases the frequency of co-infected birds, without the need to ensure frequent transmission of the co-infecting strains concurrently.

There are a number of limitations to this study. The model is stochastic and the results have only been compared to data from a single year and geographical region. Therefore, there is a chance that some parameter sets performed better than others as a consequence of stochastic variation. However, the broader finding that regular external seeding of infection into LBMs is required to generate the epidemics of multiple strains observed in surveillance data is likely to hold true for other settings. We may have underestimated the CCS for the co-infecting strains as we assumed that co-infected birds only transmitted both strains concurrently. However, the probability of transmission of each strain from a co-infected host may differ, which may reduce the frequency with which co-infection events occur increasing the CCS.

In reality more strains and host species are present within a market than modelled here, and susceptibility to infection is likely to vary between hosts and strains. We assumed there was no long-term cross-protective immunity following recovery from infection with a single strain [22], and that short-term cross-immunity was 100%. However, given the short average stay times of birds within the market system modelled, our assumption should not impact our findings on AI persistence. Within the system modelled there is competition for susceptible hosts, therefore if there were marked differences in fitness between the two strains it is possible that one strain may outcompete the other. The environmental transmission parameters were selected based on estimates from experimental data and values previously used in modelling studies, however, there remains large uncertainty in these values [3,21,23]. The impact of variation in four key parameters on the probability of persistence was done for a single strain, it would also be interesting to assess how the probability of co-infection persistence varied according to the relative combinations of these parameters across the two strains.

Many of the fundamental epidemiological features of LBMs remain poorly understood. These factors include: the physical layouts of the markets, the holding capacity of different markets, how far birds travel to come to market, how long different types of birds stay in the market, and the rate and frequency with which birds are sold onwards from the market [33,34]. Furthermore, the actual size of the market from which the data analysed here was collected remains unknown, but is likely to impact the dynamics of infection, as demonstrated by our analysis.

The trade of live birds in and between different LBMs is of cultural importance in mainland China [2], in addition to other regions in South East Asia and some parts of Africa. Periodic closure of LBMs can halt the transmission of AI, particularly with respect to preventing new human cases [35], however, in the absence of routine cleaning, market rest days and ensuring that birds come into the market infection free, wLBMs will continue to amplify AI. The simultaneous persistence of multiple strains within the market increases the probability that a bird will become co-infected. When co-infection risk is high, the risk of reassortment is increased, highlighting the important role that LBMs may play in the emergence of novel influenza A lineages. Our results demonstrate the importance of controlling the size of the market population to prevent self-sustaining transmission, but that uncertainty remains in terms of characterizing the ecology of LBMs in China.

## Supplementary Material

Supplementary material: The persistence of multiple strains of avian influenza in live bird markets

## Data accessibility

Data and code are available through Dryad (http://dx.doi.org/10.5061/dryad.np4pm) [36].
