# Supplementary material for: The persistence of multiple strains of avian influenza in live bird markets
Source: Proc Biol Sci. 2017 Dec 6;284(1868):20170715. doi: 10.1098/rspb.2017.0715 (PMC5740266; doi:10.1098/rspb.2017.0715)
Supplement: Supplementary material: The persistence of multiple strains of avian influenza in live bird markets [file rspb20170715supp1.pdf]

Supplementary material: The persistence of multiple strains of avian influenza in live bird markets

Model flow chart parameters and state variables

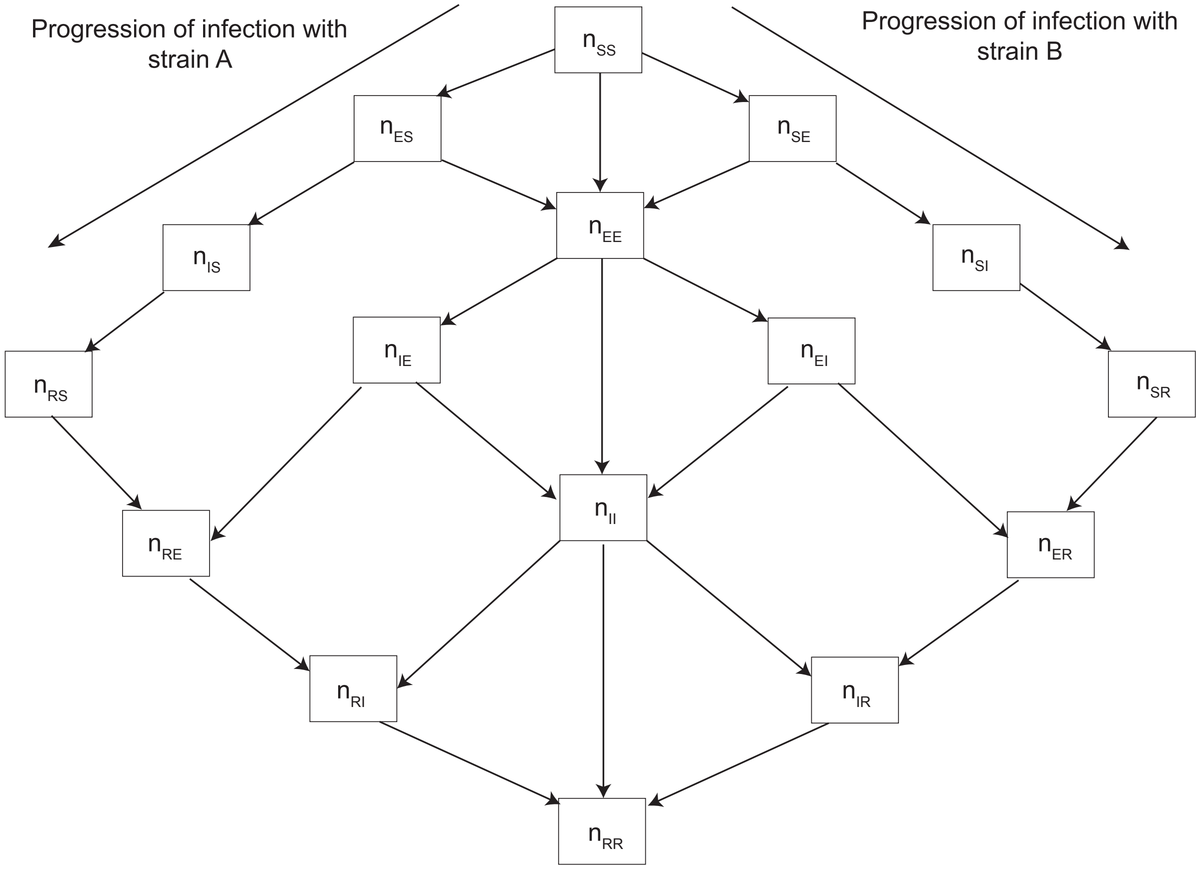

Figure S1: Flow diagram of the 2-strain SEIR co-infection model structure. Solid arrows indicate flow through the infection process once a bird becomes infected. V_A virions are shed by birds in the following states: n_{IS}, n_{IR}, n_{IE}. V_B virions are shed by birds in the states: n_{SI}, n_{RI}, n_{EI}. V_{AB} virions are shed only by birds in the n_{II} state.

Table S1 provides the baseline parameter values used in the simulation model for the estimation of the Critical Community Size (CCS).

Table S1: Initial conditions of state variables and parameter definitions used in the 2-strain within market transmission model.

Symbol	Definition	Value	Units	Source
n_{SS}	Initial number of susceptible birds to both strains	10,000	bird	
n_{ES}	Initial number of birds exposed to strain A and susceptible to B	-	bird	
n_{IS}	Initial number of birds infectious with strain A and temporally immune to B	-	bird	
n_{RS}	Initial number of birds recovered from strain A and susceptible to strain B	-	bird	
n_{SE}	Initial number of birds susceptible to strain A and exposed to strain B	-	bird	
n_{SI}	Initial number of birds immune to strain A and infectious with B	-	bird	
n_{SR}	Initial number of birds susceptible to strain A and recovered from strain B	-	bird	
n_{EE}	Initial number of birds exposed to strain A and B	-	bird	
n_{II}	Initial number of birds infectious with strain A and B	-	bird	
n_{RE}	Initial number of birds recovered from strain A and exposed to strain B	-	bird	
n_{RI}	Initial number of birds recovered from strain A and infectious with strain B	-	bird	
n_{ER}	Initial number of birds exposed to strain A and recovered from strain B	-	bird	
n_{IR}	Initial number of birds infectious with strain A and recovered from strain B	-	bird	
n_{RR}	Initial number of birds recovered from strain A and B	-	bird	
V_A	Concentration of environmental virions infectious with strain A	-	particles	
V_B	Concentration of environmental virions infectious with strain B	-	particles	
V_{AB}	Concentration of environmental virions infectious with strain AB	-	particles	
$\beta^{wf_{\{A,B,AB\}}}$	Transmission rate between birds	0.5 (0.2 - 1.5)	bird day^{-1}	$[1, 2]$
$\beta^{env_{\{A,B,AB\}}}$	Transmission rate from the environment to birds	0.17 (0.056 - 0.41)	day^{-1}	
$\sigma_{\{A,B,AB\}}$	Mean latent period (same for both strains)	1	day	$[3, 4]$
$\frac{1}{(\sigma_{\{A,B,AB\}}/n_c)}$	Rate birds leave the E compartments		day^{-1}	
$\gamma_{\{A,B,AB\}}$	Mean infectious period (same for both strains)	5	days	$[5, 6, 7, 3]$
$\frac{1}{(\gamma_{\{A,B,AB\}}/n_c)}$	Rate birds leave the I compartments		days^{-1}	
n_c	Number of compartments for exposed and infectious periods	4		
ω	Rate infected birds excrete virus to the environment	0.005 (0.0016 - 0.01)	day^{-1}	[8]
η	Rate of viral decay in the environment	0.75 (0.1 - 1.3)	day^{-1}	$[9, 10, 11]$
ψ	Restricted half-saturation constant for environmental transmission	10	particles	$[12, 13, 14]$
N_{birds}	Market holding capacity	$\sim$ 10,000	bird	
Δt	Time step	0.1	day	
ϵ	Rate birds are sold from the market	log (2)	day^{-1}	

Model description and equations

The latent period and duration of infectiousness were described by a gamma distribution implemented as the sum of four exponential distributions [15]. The mean latent period was 1 day [3,4], and 99% of birds had passed through the E compartments after 2.4 days. The mean infectious period was 5 days [5,3], and 99% of birds had passed through the I compartments after 12.6 days. The total number of birds transitioning from each compartment at any given time step Δt were sampled from the binomial distribution. When more than one transition was possible, the numbers that moved to each compartment were drawn from a multinomial distribution according to each hazards relative contribution to the overall hazard of leaving the compartment. Equivalent deterministic model equations are provided in the supplementary information.

The equivalent 2-strain deterministic model of disease transmission were governed by the following system of differential equations (in the absence of buying and selling). The first subscript denotes the progression of infection with strain A and the second denotes progression of infection with strain B : Equation 1, reflects S birds becoming infected. Equations 2 to 4 represent the progression of infection with strain A.

$$\frac{dn_{SS}}{dt} = -(\lambda^A + \lambda^B + \lambda^{AB})n_{SS} \tag{1}$$

$$\frac{dn_{ES}}{dt} = \lambda^A n_{SS} - (\lambda^B n_{ES} + \frac{1}{\sigma_A} n_{ES}) \tag{2}$$

$$\frac{dn_{IS}}{dt} = \frac{1}{\sigma_A} n_{ES} - (\frac{1}{\gamma_A} n_{IS}) \tag{3}$$

$$\frac{dn_{RS}}{dt} = \frac{1}{\gamma_A} n_{IS} - (\lambda^B n_{RS}) \tag{4}$$

Here λ^A is defined as:

$$\lambda^A(t) = \beta^{wf,A}\left(\frac{\big(n_{IS}(t) + n_{IE}(t) + n_{IR}(t)\big)}{N(t)}\right) + \beta^{A_{env}}\left(\frac{V_A(t)}{V_A(t) + \psi}\right), \tag{5}$$

Here $\beta^{wf,A}$ was the transmission parameter, which was the probability that contact with an infectious bird resulted in successful onwards transmission of infection to another bird. $n_{IS}(t)+n_{IE}(t)+n_{IR}(t)$ was the total number of infectious birds with strain A in the market, at each time point. This was divided by the total population size of the market $N(t)$. For the environmental transmission component β^{env} was the probability that an infectious virion in the environment successfully infected a bird. $V_A(t)$ was the concentration of infectious virions of strain A in the environment at time t and ψ was the concentration of virions that resulted in 50% of the maximum infection rate [14, 13, 12]. Virions entered the environment as they were exhaled or excreted by infectious birds, at a rate ω and decayed at a constant rate η. Similarly λ^B for the B is:

$$\lambda^B(t) = \beta^{wf,B}\left(\frac{\left(n_{SI}(t)+n_{EI}(t)+n_{RI}(t)\right)}{N(t)}\right) + \beta^{B_{env}}\left(\frac{V_B(t)}{V_B(t)+\psi}\right), \tag{6}$$

and λ^{AB}:

$$\lambda^{AB}(t) = \beta^{wf,AB}\left(\frac{n_{II}(t)}{N(t)}\right) + \beta^{AB_{env}}\left(\frac{V_{AB}(t)}{V_{AB}(t)+\psi}\right) \tag{7}$$

Therefore the force of infection for each strain consisted of two components. The first was the within-flock force of infection representing direct bird-to-bird transmission, modelled as a frequency-dependent process. The second was the environmental transmission component which was modelled as a density-dependent process. As bird-to-bird transmission was modelled as a frequency-dependent process, we assumed a bird's ability to infect a fixed number of birds was not affected by increases or decreases in the size of the market population. This assumption implied that the direct transmission potential of infected birds was restricted, and in reality may reflect the movement restrictions birds experience within the market, as they are likely to be caged up or crammed within a pen. This contact rate assumption is supported by experimental work [2]. Environmental transmission was modelled as a density-dependent process. Dispersal of infectious matter is likely to be less spatially restricted than

bird-to-bird contact, and the spread of fomites has been shown to occur through the air and wind dispersal [16, 17]. In addition, unlike markets in Hong Kong where cleaning and closure of the markets is done on a routine basis [18, 19], the vast majority of rural markets are rarely if ever cleaned. The assumption of density-dependent environmental transmission may be unrealistic because the environment itself will have a maximum capacity in terms of the concentration of virions it can maintain. However it seems reasonable that the true mechanism of transmission in the market could be described by a mix of frequency and density-dependent transmission. Neither of the ways in which transmission has been modelled are likely to describe the system perfectly, however the inclusion of both mechanisms may capture the variation inherent in the system.

Equations 8 to 10 represent the progression of infection with strain B.

$$\frac{dn_{SE}}{dt} = \lambda^B n_{SS} - (\lambda^A n_{SE} + \frac{1}{\sigma_B} n_{SE}) \tag{8}$$

$$\frac{dn_{SI}}{dt} = \frac{1}{\sigma_B} n_{SE} - (\frac{1}{\gamma_B} n_{SI}) \tag{9}$$

$$\frac{dn_{SR}}{dt} = \frac{1}{\gamma_B} n_{SI} - (\lambda^A n_{SR}) \tag{10}$$

Equations 11 to 14 represent birds who have been co-infected with both strains. They can be exposed to both, infectious with both, or exposed to one and infectious with the other.

$$\frac{dn_{EE}}{dt} = (\lambda^{AB} n_{SS} + \lambda^B n_{ES} + \lambda^A n_{SE}) - (\frac{1}{\sigma_B} n_{EE} + \frac{1}{\sigma_A} + n_{EE}) \tag{11}$$

$$\frac{dn_{IE}}{dt} = \frac{1}{\sigma_A} n_{EE} - (\frac{1}{\gamma_A} n_{IE} + \frac{1}{\sigma_B} n_{IE}) \tag{12}$$

$$\frac{dn_{EI}}{dt} = \frac{1}{\sigma_B} n_{EE} - (\frac{1}{\sigma_A} n_{EI} - \frac{1}{\gamma_B} n_{EI}) \tag{13}$$

$$\frac{dn_{II}}{dt} = (\frac{1}{\sigma_A} + \frac{1}{\sigma_B}) - (\frac{1}{\gamma_B} n_{II} + \frac{1}{\gamma_A} n_{II}) \tag{14}$$

Birds that have recovered from infection with strain A and have been infected with strain

B.

$$\frac{dn_{RE}}{dt} = (\lambda^B n_{RS} + \frac{1}{\gamma_A} n_{IE}) - (\frac{1}{\sigma_B} n_{RE}) \quad (15)$$
$$\frac{dn_{RI}}{dt} = (\frac{1}{\sigma_B} n_{RE} + \frac{1}{\gamma_A} n_{II}) - (\frac{1}{\gamma_B} n_{RI}) \quad (16)$$
$$\frac{dn_{ER}}{dt} = (\lambda^A n_{SR} + \frac{1}{\gamma_A} n_{EI}) - (\frac{1}{\sigma_A} n_{ER}) \quad (17)$$
$$\frac{dn_{IR}}{dt} = (\frac{1}{\sigma_A} n_{ER} + \frac{1}{\gamma_B} n_{II}) - (\frac{1}{\gamma_B} n_{IR}) \quad (18)$$
$$\frac{dn_{RR}}{dt} = \frac{1}{\gamma_B} n_{RI} + \frac{1}{\gamma_A} n_{IR} \quad (19)$$

The last set of equations model the concentration of virions of each strain in the environment.

$$\frac{dV_A}{dt} = -\eta V_A + \omega(n_{IS} + n_{IE} + n_{IR}) \quad (20)$$
$$\frac{dV_B}{dt} = -\eta V_B + \omega(n_{SI} + n_{EI} + n_{RI}) \quad (21)$$
$$\frac{dV_{AB}}{dt} = -\eta V_{AB} + \omega n_{II} \quad (22)$$

The model described by the differential equations 1 to 19 and presented in Figure S1 was implemented in a stochastic framework. The concentration of virons in equations 20 to 22 were modelled deterministically.

Derivation of R_0 for a single strain model

The R_0 for the single strain market model which included both within flock transmission and environmental transmission was calculated as follows.

The within flock component of R_0 (R_0^{wf}) for the $SEIR$ single strain model in the absence of environmental transmission is as follows:

Firstly, we need to account for the probability that birds make it through each of the 4 E compartments without being sold, and thus progress to the infectious state. The probability of making it through a single E compartment is:

$$\frac{4\sigma}{4\sigma + \epsilon} \tag{23}$$

therfore the probability that a bird makes it through all 4 compartments is:

$$\left(\frac{4\sigma}{4\sigma + \epsilon}\right)^4 \tag{24}$$

The duration of time a bird spends in the first I compartment is:

$$\frac{1}{4\gamma + \epsilon} \tag{25}$$

hence, for the 2nd I compartment we consider the probability a bird makes it through the first I compartment and the duration of time spent in the second compartment:

$$\frac{4\gamma}{4\gamma + \epsilon} + \frac{1}{4\gamma + \epsilon} \tag{26}$$

thus, for the third I compartment we get:

$$\left(\frac{4\gamma}{4\gamma + \epsilon}\right)^2 + \frac{1}{4\gamma + \epsilon} \tag{27}$$

and for the fourth I compartment we get:

$$\left(\frac{4\gamma}{4\gamma+\epsilon}\right)^3 + \frac{1}{4\gamma+\epsilon} \tag{28}$$

Summing this geometric series this simplifies to:

$$\frac{1}{\epsilon}\left(1-(\frac{4\gamma}{4\gamma+\epsilon})^4\right) \tag{29}$$

therefore the within flock R_0 is:

$$R_0^{wf} = \left(\frac{4\sigma}{(4\sigma+\epsilon)}\right)^4 \frac{1}{\epsilon}\left(1-\frac{4\gamma}{(4\gamma+\epsilon)}\right)^4 \beta^{wf} \tag{30}$$

The first term reflects the probability that a bird progresses from infected to infectious whilst still in the market. The second term accounts for the time spent in the I state where a bird can transmit infection. β^{wf} is the transmission rate between an infectious and susceptible bird.

We then need to calculate the contribution to R_0 from environmental transmission, denoted R_0^{env}. To do this, we first consider a situation where the I is represented by only 1 compartment (this comparably more simple derivation is presented for illustratory purposes), therefore we can look at the expected number of virions excreted by a single bird over the course of infection. Starting with only one bird in the E state, zero birds in the I, a wholly susceptible population and a clean environment, we can calculate the amount of time spent infected by solving the following:

$$\frac{dE}{dt} = -\sigma E - \epsilon E \tag{31}$$

$$\frac{dI}{dt} = \sigma E - \gamma I - \epsilon I \tag{32}$$

Equations 31 and 32 can be solved analytically to give:

$$I(t) = \frac{\sigma}{\sigma - \gamma}\big(e^{-(\gamma+\epsilon)t} - e^{-(\sigma+\epsilon)t}\big) \tag{33}$$

We can then assess how many virions are excreted by this single infected bird by solving:

$$\frac{dV_1}{dt} = \omega\left(\frac{\sigma}{\sigma - \gamma}\big(e^{-(\gamma+\epsilon)t} - e^{-(\sigma+\epsilon)t}\big)\right) - \eta V_1 \tag{34}$$

This can be solved analytically (again under the assumption of a 1 compartment model) to give:

$$V_1(t) = \frac{\sigma\omega\big(\gamma - \sigma\big)e^{-\eta t} + \sigma\omega\big(\epsilon + \sigma - \eta\big)e^{-(\gamma+\epsilon)t} - \sigma\omega\big(\gamma + \epsilon - \eta\big)e^{(\sigma+\epsilon)t}}{(\gamma - \sigma)(\gamma + \epsilon - \eta)(\epsilon + \sigma - \eta)} \tag{35}$$

Knowing the number of virions in the environment over time, we can calculate the probability of a bird becoming infected as follows:

$$P^{env} = 1 - e^{\int_0^\infty \beta^{env} \frac{V_1(t)}{V_1(t)+\psi} dt}, \tag{36}$$

under the assumption that the number of virions from a single bird is very small ($V_1 \ll$

ψ), we can make some simplifying assumptions:

$$\frac{V_1}{V_1 + \psi} \approx \frac{V_1}{\psi} \tag{37}$$

and hence using the Taylor expansion, (where $e^x \approx 1 + x$ for small x) we obtain:

$$P^{env} = 1 - e^{\int_0^\infty \beta^{env} \frac{V_1(t)}{\psi} dt} \approx \int_0^\infty \beta^{env} \frac{V_1(t)}{\psi} dt \tag{38}$$

We can therefore calculate the total number of environmental transmissions as follows:

$$R_0^{env} = N \int_0^\infty \beta^{env} \frac{V_1(t)}{\psi} dt \tag{39}$$

$$= \left(\frac{\sigma}{(\sigma + \epsilon)}\right) \frac{\omega N}{\eta \psi} \frac{1}{\epsilon} \left(1 - \frac{\gamma}{(\gamma + \epsilon)}\right) \beta^{env} \tag{40}$$

Therefore by extension if we then consider the four compartmental model (as assumed throughout the manuscript) the environmental R_0 is proportional to the time spent secreting virus, hence R_0^{env} is:

$$R_0^{env} = N \int_0^\infty \beta^{env} \frac{V_1(t)}{\psi} dt \tag{41}$$

$$= \left(\frac{4\sigma}{(4\sigma + \epsilon)}\right)^4 \frac{\omega N}{\eta \psi} \frac{1}{\epsilon} \left(1 - \frac{4\gamma}{(4\gamma + \epsilon)}\right)^4 \beta^{env} \tag{42}$$

Where the first term considers the probability that a bird makes it to the I state while still in the market. The second term is a measure for the concentration of virions in the environment, hence we consider the ratio of production ω to decay η, N reflects the density-dependence of environmental transmission and ψ is the concentration of virions that results in 50% of the maximum infection rate. The third term is the mean time spent in the I state

while a bird is in the market and β^{env} is the transmission rate between the environment and a susceptible bird.

and finally, the total basic reproduction number was:

$$R_0 = R_0^{wf} + R_0^{env} \tag{43}$$

Selecting an appropriate seed size for CCS estimation

The CCS was estimated through simulation. Infection within the market was seeded for 1 day with 25 birds for the single strain analysis and 50 birds (25 of each strain) for the co-infection analysis. 1,000 stochastic realisations were run for each N for 365 days to calculate the fadeout fraction.

We calculated the invasion probability for an R_0 of 1.15 across a range of different seed sizes (from 1-50 seeds) for a single , as shown in Figure S3. Invasion probability for a fixed value of R_0 and a given seed size was performed according to the method outlined in [20]. We therefore selected a seed size of 25 as this ensured a high probability of invading.

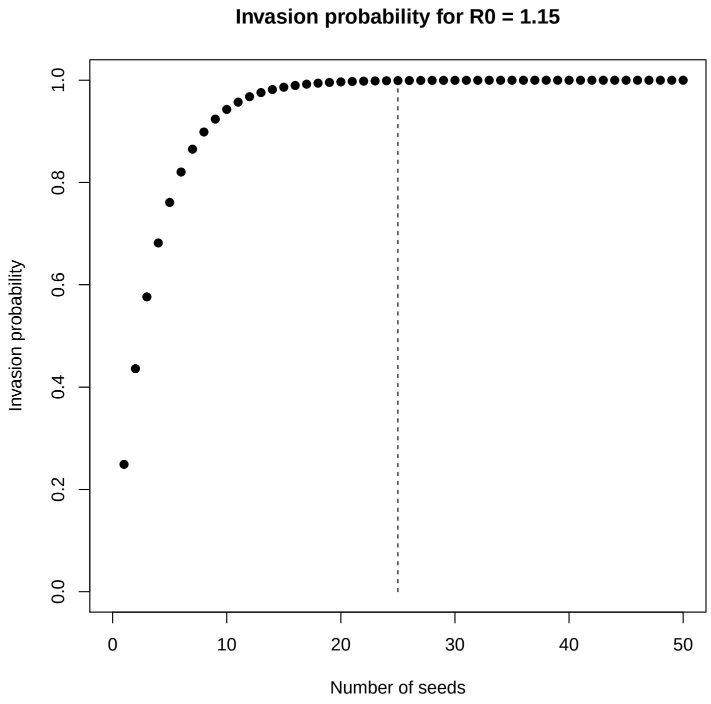

Figure S2: The invasion probability for a single strain across a range of seed sizes, from 1 - 50 seeds. We use this curve to determine an appropriate number of seeds to initialise the CCS simulations.

Seeding models evaluated for the 2-strain model

R_0 for a single strain

Estimates of R_0 for each scenario analysed to estimate the CCS (Table S3).

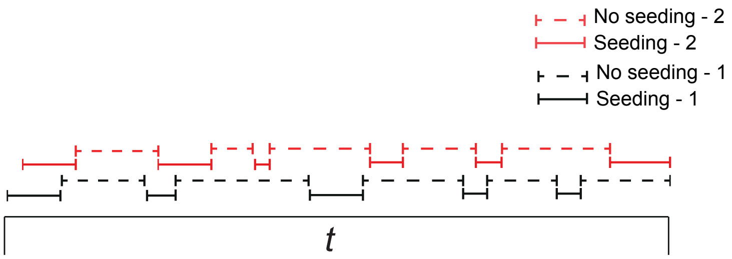

Figure S3: Schematic of infection seeding scenarios. Infection was seeded into the market over short periods of time followed by slightly longer periods where infection was not seeded. The two colours (black and red) indicate that the seeding of the two different s occurred at different times.

Sensitivity analysis of parameters influencing the probability of persistence of a single strain

We investigated how variation in 4 key assumed epidemiological parameters altered the probability of persistence for a single strain within our model. We performed a latin hypercube sample of 1000 different parameter sets for each of the 4 different market population sizes selected and calculate the probability of persistence for each parameter set after 500 stochastic realisations. We then calculated the partial rank correlation coefficient (PRCC) for each parameter to quantify the significance of their impact on the outcome variable (in our case the probability of persistence of a single strain). We calculated the standard deviation of the mean for probability of persistence for each range of parameter values provided, we used +/- the standard deviation of the mean to calculate the uncertainty about the mean for all parameters, this uncertainty is presented as a polygon in each figure (Figure 2). We then calculated the partial rank correlation coefficient for each parameter and the outcome variable for the 4 different population sizes tested, results are presented in Tables S4 and S5.

We initially plotted the relationship between the fade-out fraction and market population size for each parameter (Figure S4).

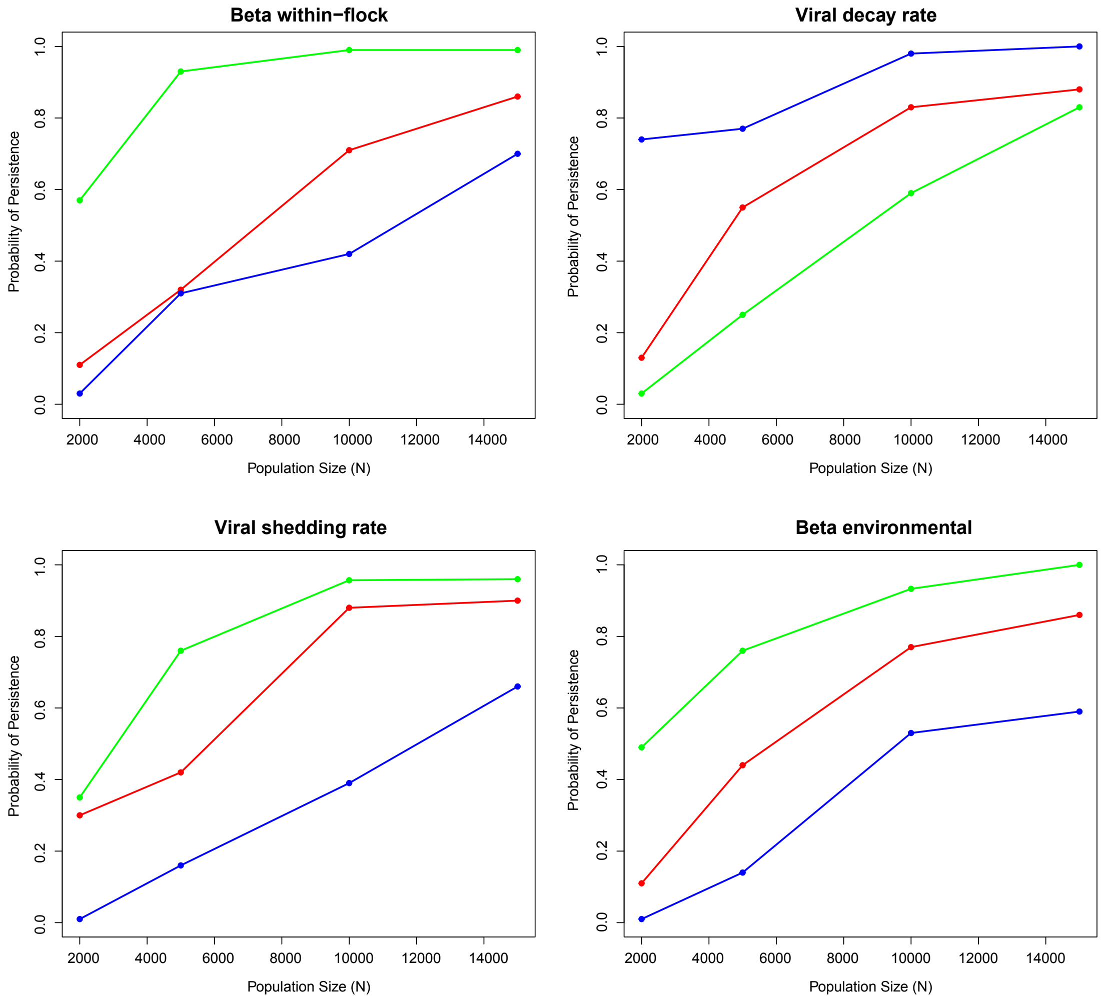

Figure S4: The relationship between the average market population size and probability of persistence for each of the four epidemiological parameters. Results using the baseline parameters are presented in red. Results using the upper values of each parameter are shown in green and lower values of each parameter are shown in blue.

Table S2: A description of each of the seeding models evaluated.

Model[1]	Prevalence range[2]	Range of seeding (days)[3]	Range of non-seeding (days)[4]
Model 1	0 - 0.7	5- 30, 5 - 15	20 - 80, 40 - 80
Model 2	0 - 0.5	5 - 30, 10 - 30	40 - 80, 20 - 80
Model 3	0 - 0.5	1 - 20, 1 - 30	20 - 80, 50 - 100
Model 4	0.2 - 0.7	5 - 10, 5 - 20	10 - 30, 15 - 30
Model 5	0.2 - 0.7	1 - 30, 1 - 20	40 - 50, 40 - 100
Model 6	0 - 0.1	1 - 5, 1 - 5	80 - 150, 80 - 150
Model 7	0 - 0.1	1 - 3, 1 - 3	80 - 200, 80 - 200
Model 8	0 - 0.02	1 - 3, 1 - 3	80 - 200, 70 - 220
Model 9	0.2 - 0.7	1 - 3, 1 - 10	2 - 20, 5 - 20
Model 10	0.02 - 0.7	4 - 12, 1 - 5	10 - 40, 15 - 30

[1] The name of each model used. [2] The range of prevalence of infection of birds brought into the market.[3] The range of days for which no seeding occurred i.e. no new infected birds were introduced into the market. The two sets of numbers represent the ranges of days used for strain A and strain B. [4] The ranges of days for which seeding did occur i.e. new infected birds were introduced into the market. The two sets of numbers represent the ranges of days used for strain A and strain B.

Persistence and CCS estimates of a single and co-infecting strains with instantaneous and gradual market turnover

Table S3: Calculated value of R_0 for each CCS scenario evaluated across all market turnover rates (single strain).

**Turnover Rate**	**Baseline - single strain**	**25% increase in single strain**	**75% increase in single strain**
10%	1.67	2.43	4.21
20%	1.16	1.59	2.53
30%	1.00	1.12	1.61
40%	1.01	1.05	1.22
50%	1.01	1.11	1.14
60%	1.26	1.22	1.32
70%	1.54	1.54	1.62
80%	1.90	2.08	1.86
90%	2.23	2.19	2.22

Table S4: Output from PRCC analysis when R_0 was constrained.

**Parameter and population size**	**PRCC**	**P-value**
N = 2000		
β^{wf}	-0.580	2.636 x 10^{-91}
η	0.835	2.516 x 10^{-262}
ω	-0.717	1.147 x 10^{-158}
β^{env}	-0.727	1.235 x 10^{-165}
N = 5000		
β^{wf}	-0.415	1.211 x 10^{-43}
η	0.715	1.130 x 10^{-157}
ω	-0.651	5.122 x 10^{-122}
β^{env}	-0.682	3.264 x 10^{-138}
N = 10000		
β^{wf}	-0.344	2.258 x 10^{-29}
η	0.586	2.291 x 10^{-93}
ω	-0.615	3.751 x 10^{-105}
β^{env}	-0.633	4.202 x 10^{-113}
N = 15000		
β^{wf}	-0.323	8.085 x 10^{-26}
η	0.500	2.988 x 10^{-64}
ω	-0.538	3.056 x 10^{-76}
β^{env}	-0.603	2.172 x 10^{-100}

Table S5: Output from PRCC analysis when R_0 was not constrained.

**Parameter and population size**	**PRCC**	**P-value**
N = 2000		
β^{wf}	-0.536	1.00 x 10^{-75}
η	0.622	3.093 x 10^{-108}
ω	-0.434	2.470 x 10^{-47}
β^{env}	-0.489	2.684 x 10^{-61}
N = 5000		
β^{wf}	-0.576	1.281 x 10^{-89}
η	0.695	9.951 x 10^{-146}
ω	-0.603	2.983 x 10^{-100}
β^{env}	-0.640	1.893 x 10^{-116}
N = 10000		
β^{wf}	-0.384	1.653 x 10^{-36}
η	0.564	2.186 x 10^{-85}
ω	-0.543	7.096 x 10^{-78}
β^{env}	-0.585	4.396 x 10^{-93}
N = 15000		
β^{wf}	-0.286	2.792 x 10^{-20}
η	0.428	6.902 x 10^{-46}
ω	-0.417	1.960 x 10^{-43}
β^{env}	-0.489	2.228 x 10^{-61}

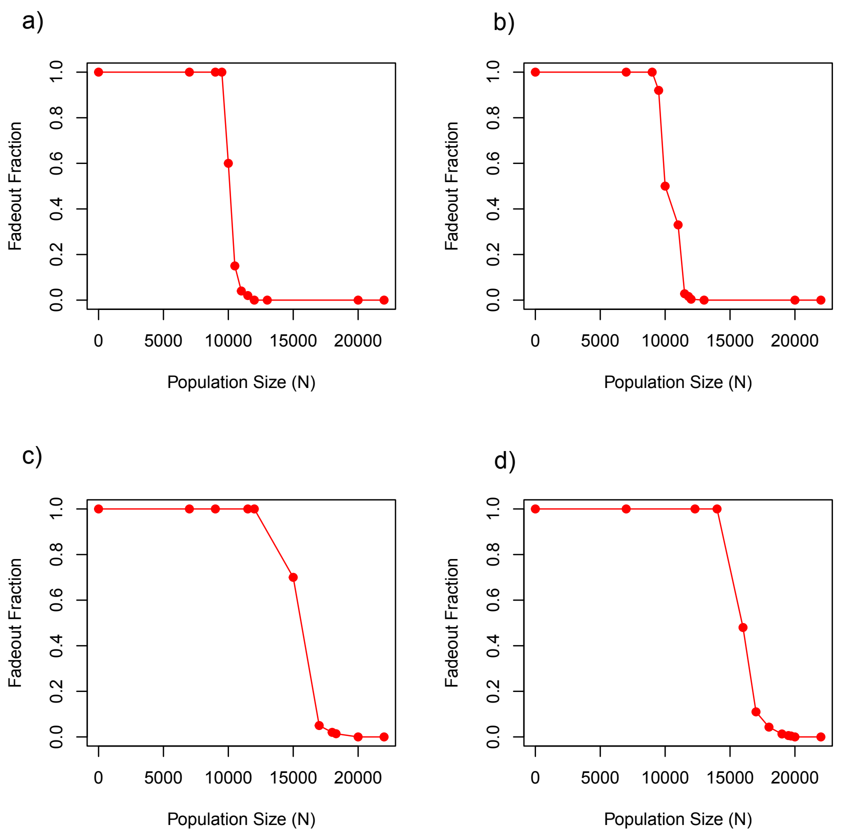

Figure S5: The CCS estimated through simulation analysis for 1000 realisations, for a range of instantaneous and gradual market turnover rates. a) For a fixed market turnover rate of 50% assuming the baseline level of transmissibility, the fade-out fraction for a single strain within the market for a range of population sizes is indicated. b) For a fixed market turnover rate of 50% assuming the baseline level of transmissibility, the fade-out fraction of the co-infecting strain within the market for a range of population sizes is indicated. c) For a gradual market turnover rate of 50% assuming the baseline level of transmissibility, the fade-out fraction for a single-strain within the market for a range of population sizes is indicated. d) For a gradual market turnover rate of 50% assuming the baseline level of transmissibility, the fade-out fraction for the co-infecting strain within the market for a range of population sizes is indicated.

Persistence of a co-infected under vary sensitivities

CCS for co-infected strain with variable market turnover and the duration of the latent period

We assessed the estimate of the CCS across a range of plausible latent periods and market turnover rates (Figure S6). This was done as it has been shown that the duration of the latent period can be vary by dose and strain. We considered a range of latent periods with a mean duration between 0.5 - 6 days. Overall, as the duration of the incubation period and the market turnover rate increased the estimated size of the CCS increased substantially.

Relationship between population size and the probability of persistence of the co-infected strain

We observed that as the probability of transmission increased for a fixed population size the probability of persistence of the co-infected strain increased (Figure S7). Equally, for a fixed level of transmissibility, shedding and decay rates as well as infection duration, as the population size increased the probability of persistence also increased.

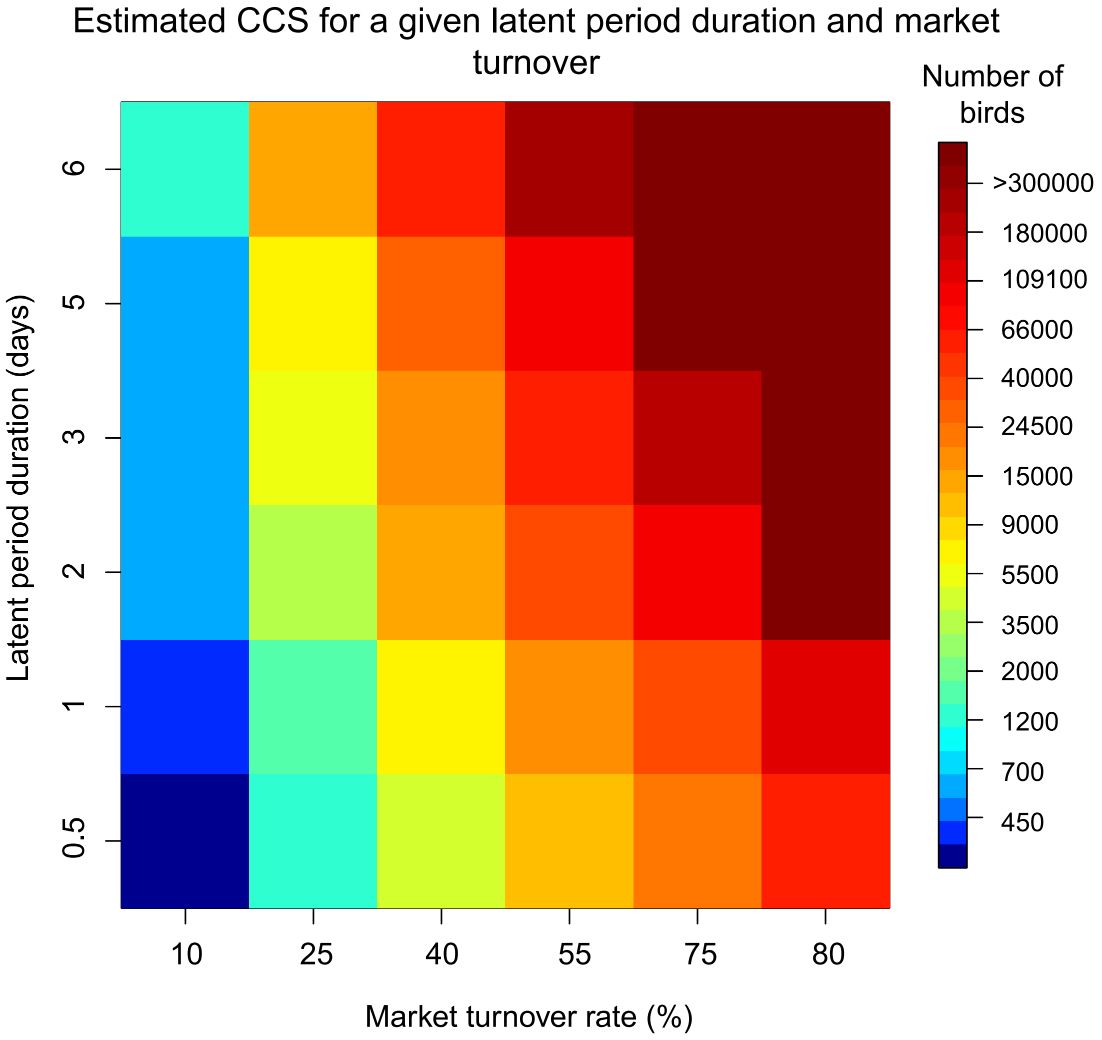

Figure S6: The estimated CCS for a range of different incubation periods and market turnover rates. The colour indicates the estimated size of the CCS. The estimated size of each CCS on the heatmap is presented as the log transform of the original value.

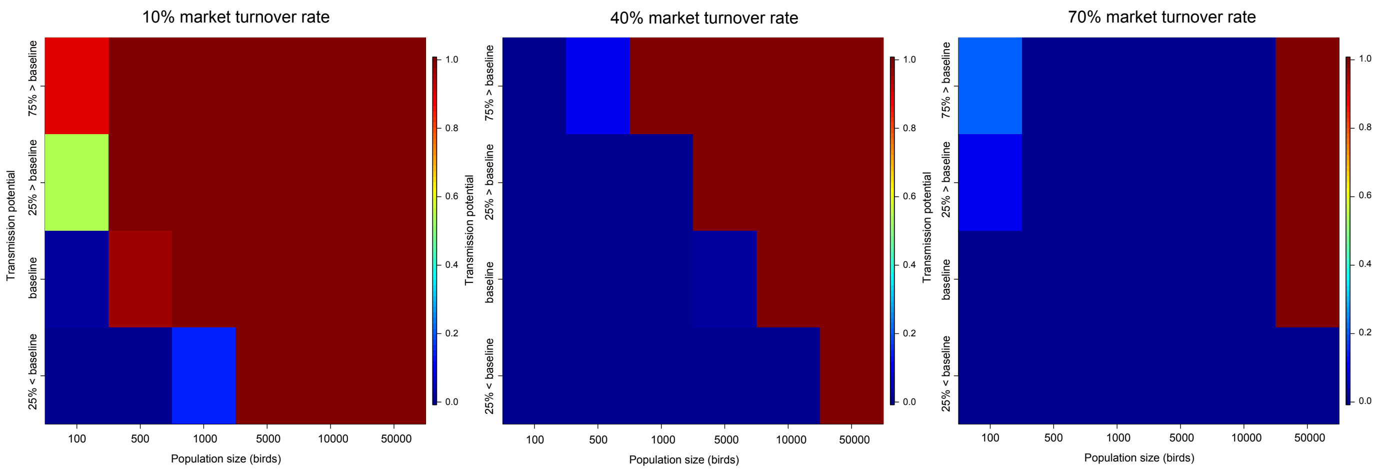

Figure S7: The probability of persistence for the co-infecting strain for a range of different population sizes, with increasing and decreasing levels of: transmissibility from other birds and the environment, shedding and decay rates as well as infection duration and market turnover rates. The colour indicates probability of fade out for each parameter set calculated from 1000 iterations.

Results from the different seeding models

We began by exploring whether the 2-strain dynamics observed in the Shantou wLBM surveillance data could be reproduced by autochthonous transmission (sustained transmission without importation). We seeded each strain for five days once only, where a fixed proportion (0.4) of birds came into the market in the I state for this time period, while the remaining birds were imported in the S state.

Comparing the results of the simulation with a single five day seed to the surveillance data (Figure S8), autochthonous transmission alone was unable to ensure that the regular outbreaks observed in the data were generated in the simulation. Therefore we explored whether random periods of seeding and non-seeding times could better replicate the observed dynamics.

While overall model performance was evaluated across all 4 summary statistics we also assessed what proportion of realisations matched across 2 and 3 of the summary statistics

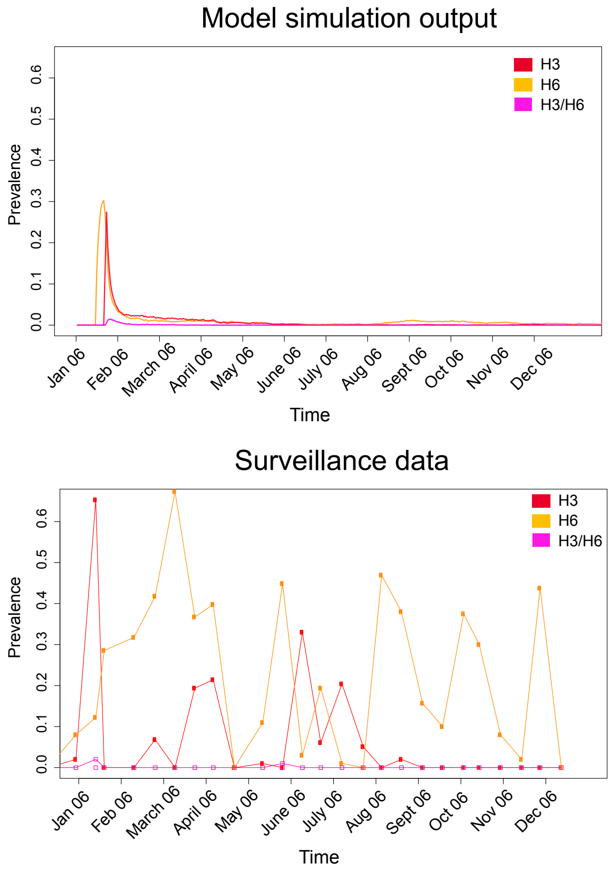

Figure S8: A single model simulation with each strain seeded for 5 days only at the start of the time series. With a single introduction of both strains at the start of the time series it was not possible to replicate the dynamics observed in the data.

evaluated, as we note some models performed well across several summary statistics but poorly across 1, resulting in the intersection across all 4 statistics being equal to 0.

Overall for models 5-10 we saw a very poor intersection between summary statistics across all combinations evaluated.

Subsets of the best performing model for the 2-strain analysis

We draw four random subsets from the best performing model and plot them against the data to assess the visual fit (Figure S9).

Table S6: Results from comparing the intersection of less than 4 summary statistics for each model

Comparison statistics[1]	Model 1	Model 2	Model 3	Model 4	Model 5	Model 6	Model 7	Model 8	Model 9	Model 10
1, 2	0.20	0.04	0.16	0	0.16	0	0	0	0	0
1, 3	0.21	0.10	0.12	0	0.11	0.001	0.003	0.001	0.001	0.06
1, 4	0.01	0.09	0.03	0	0.03	0	0	0	0	0
2, 3	0.12	0.02	0.06	0	0.10	0	0	0	0	0
2, 4	0.03	0.05	0	0	0.06	0	0	0	0	0
3, 4	0.01	0.05	0.01	0	0.02	0	0	0	0	0
1, 2, 4	0.017	0.025	0	0.03	0	0	0	0	0	0
1, 3, 4	0.006	0.002	0.007	0	0.02	0	0	0	0	0
1, 2, 3	0.08	0.01	0.04	0	0.05	0	0	0	0	0
2, 3, 4	0.009	0.013	0	0	0.02	0	0	0	0	0

[1] The summary statistics being compared. Where 1 = the correlation between the two strains, 2 = the mean prevalence of the 2 strains, 3 = the fourier transform and 4 = the number of peaks of the two strains.

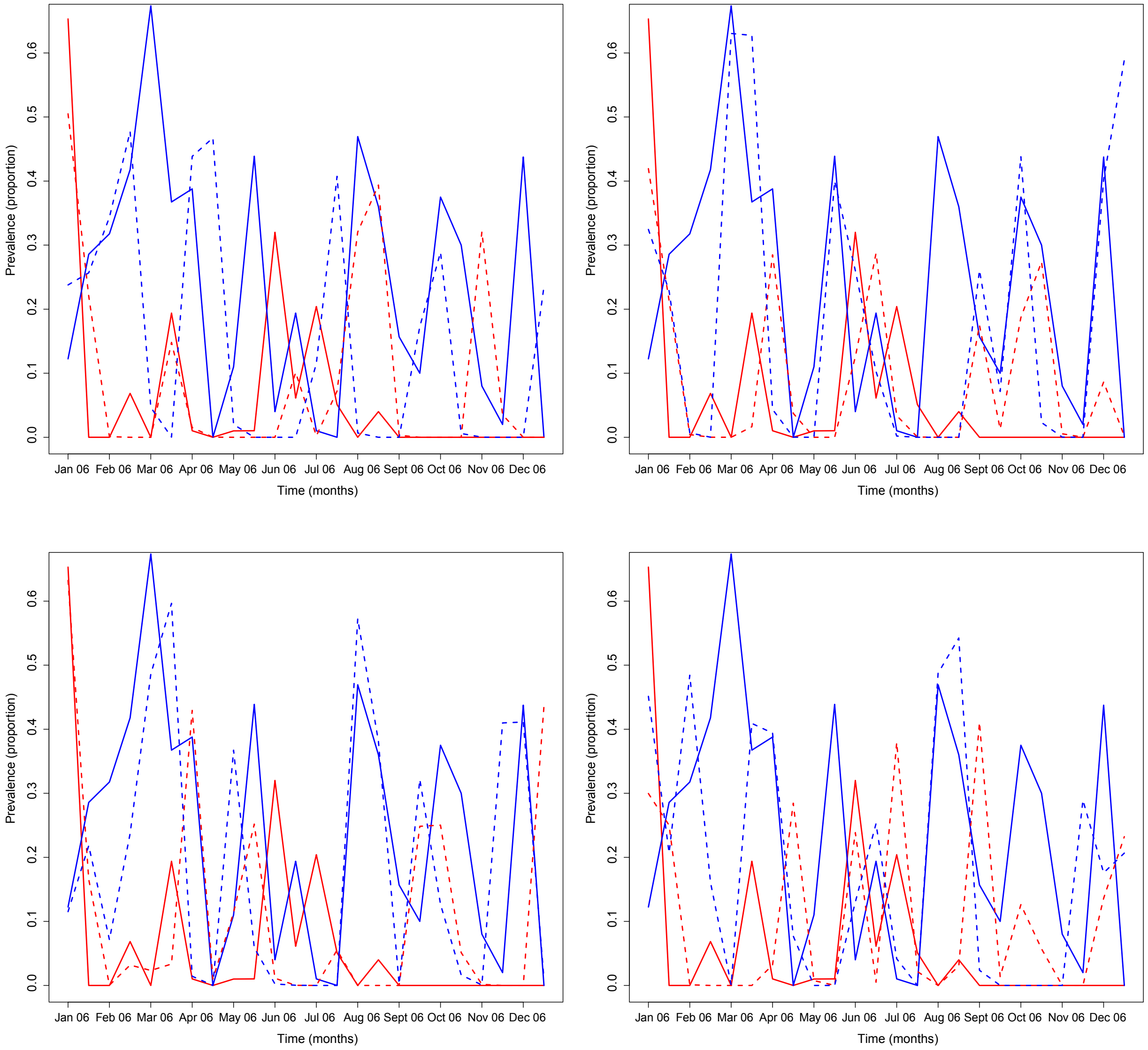

Figure S9: A subset of four stochastic realisations from the best performing seeding model, identified in Table 1. Red and orange indicate the two co-circulating strains. Solid line are the data and dashed lines are the simulation output. We note that while not every realisation fits visually well to the data, this model was selected on the basis that it was able to capture the summary statistics derived from the data and hence captures the key epidemiological features of the system.

References

[1] A. Stegeman, A. Bouma, A. Elbers, M. De Jong, G. Nodelijk, F. de Klerk, G. Koch, and M. Van Boven, "Avian Influenza A Virus (H7N7) Epidemic in The Netherlands in 2003: Course of the Epidemic and Effectiveness of Control Measures," *The Journal of Infectious Diseases*, vol. 190, no. 12, pp. 2088 – 2095, 2004.

[2] R. A. Saenz, S. C. Essen, S. M. Brookes, M. Iqbal, J. L. N. Wood, B. T. Grenfell, J. W. McCauley, I. H. Brown, and J. R. Gog, "Quantifying transmission of highly pathogenic and low pathogenicity h7n1 avian influenza in turkeys," *PLOS ONE*, vol. 7, no. 9, pp. 1–9, 09 2012.

[3] M. Iqbal, T. Yaqub, N. Mukhtar, M. Shabbir, and J. McCauley, "Infectivity and transmissibility of H9N2 avian influenza virus in chickens and wild terrestrial birds," *Veterinary Research*, vol. 44, no. 1, 2013.

[4] J. Post, E. de Geus, L. Vervelde, J. Cornelissen, and J. Rebel, "Systemic distribution of different low pathogenic avian influenza (LPAI) viruses in chicken," *Virology Journal*, vol. 10, no. 23, 2013.

[5] A. Comin, D. Klinkenberg, S. Marangon, A. Toffan, and A. Stegeman, "Transmission Dynamics of Low Pathogenicity Avian Influenza Infections in Turkey Flocks," *PLoS ONE*, vol. 6, no. 10, p. e26935, 2011.

[6] M. J. Pantin-Jackwood, P. J. Miller, E. Spackman, D. E. Swayne, L. Susta, M. Costa Hurtado, and D. L. Suarez, "Role of poultry in spread of novel H7N9 influenza virus in China," *Journal of Virology*, vol. 88, no. 10, pp. 5381 – 5390, 2014.

[7] T. Costa, J. Brown, E. Howerth, and D. Stallknecht, "The effect of age on avian influenza viral shedding in mallards (*Anas platyrhynchos*)," *Avian Diseases*, vol. 54, no. Suppl 1, pp. 581 – 585, 2010.

[8] R. Breban, J. M. Drake, D. E. Stallknecht, and P. Rohani, "The Role of Environmental Transmission in Recurrent Avian Influenza Epidemics," *PLoS Computational Biology*, vol. 5, no. 4, p. e1000346, 2009.

[9] Y. Leung, L. Zhang, C. Chow, C. Tsang, C. Ng, C. Wong, Y. Guan, and J. Peiris, "Poultry drinking water used for avian influenza surveillance," *Emerging Infectious Diseases*, vol. 13, no. 9, pp. 1380 – 1382, 2007.

[10] A. Handel, J. Brown, D. Stallknecht, and P. Rohani, "A multi-scale analysis of influenza a virus fitness trade-offs due to temperature-dependent virus persistence," *PLOS Computational Biology*, vol. 9, no. 3, pp. 1–13, 2013.

[11] A. Handel, C. Lebarbenchon, D. Stallknecht, and P. Rohani, "Trade-offs between and within scales: environmental persistence and within-host fitness of avian influenza viruses," *Proceedings of the Royal Society of London B: Biological Sciences*, vol. 281, no. 1787, p. 20133051, 2014.

[12] P. Rohani, R. Breban, D. E. Stallknecht, and J. M. Drake, "Environmental transmission of low pathogenicity avian influenza viruses and its implications for pathogen invasion," *Proceedings of the National Academy of Sciences*, vol. 106, no. 25, pp. 10 365 – 10 369, 2009.

[13] B. Roche and P. Rohani, "Environmental transmission scrambles coexistence patterns of avian influenza viruses," *Epidemics*, vol. 2, no. 2, pp. 92 – 98, 2010.

[14] K. M. Pepin, J. O. Lloyd-Smith, C. T. Webb, K. Holcomb, H. Zhu, Y. Guan, and S. Riley, "Minimizing the threat of pandemic emergence from avian influenza in poultry systems," *BMC Infectious Diseases*, vol. 13, no. 592, 2013.

[15] H. Wearing, P. Rohani, and M. Keeling, "Appropriate Models for the Management of Infectious Diseases," *PLoS Medicine*, vol. 2, no. 7, p. e174, 2005.

[16] R. J. Ypma, M. Jonges, A. Bataille, A. Stegeman, G. Koch, M. van Boven, M. Koopmans, W. M. van Ballegooijen, and J. Wallinga, "Genetic data provide evidence for wind-mediated transmission of highly pathogenic avian influenza," *The Journal of Infectious Diseases*, vol. 207, no. 5, p. 730, 2013.

[17] M. Jonges, J. van Leuken, I. Wouters, G. Koch, A. Meijer, and M. Koopmans, "Wind-mediated spread of low-pathogenic avian influenza virus into the environment during outbreaks at commercial poultry farms," *PLOS ONE*, vol. 10, no. 5, pp. 1–15, 2015.

[18] E. Lau, Y. Leung, L. Zhang, B. Cowling, S. Mak, Y. Guan, G. Leung, Peiris, and JS, "Effect of interventions on influenza A (H9N2) isolation in Hong Kongs live poultry markets, 1999 - 2005," *Emerging Infectious Diseases*, vol. 13, no. 9, pp. 1340 – 1347, 2007.

[19] Y. Leung, E. Lau, L. Zhang, Y. Guan, B. Cowling, and J. Peiris, "Avian Influenza and Ban on Overnight Poultry Storage in Live Poultry Markets, Hong Kong," *Emerging Infectious Diseases*, vol. 18, no. 8, pp. 1339 – 1341, 2007.

[20] W. M. Getz and J. O. Lloyd-Smith, "Basic methods for modeling the invasion and spread of contagious diseases," in *Disease Evolution: Models, Concepts, and Data Analyses.* Citeseer, 2006, pp. 87–112.
